# Supplementary material for: Molecular basis of Tousled-Like Kinase 2 activation
Source: Nat Commun. 2018 Jun 28;9:2535. doi: 10.1038/s41467-018-04941-y (PMC6023931; doi:10.1038/s41467-018-04941-y)
Supplement: Supplementary file 1 — Supplementary Information [file 41467_2018_4941_MOESM1_ESM.pdf]

Molecular Basis of Tousled-like kinase 2 activation. Mortuza and Hermida et al.

(Section A)

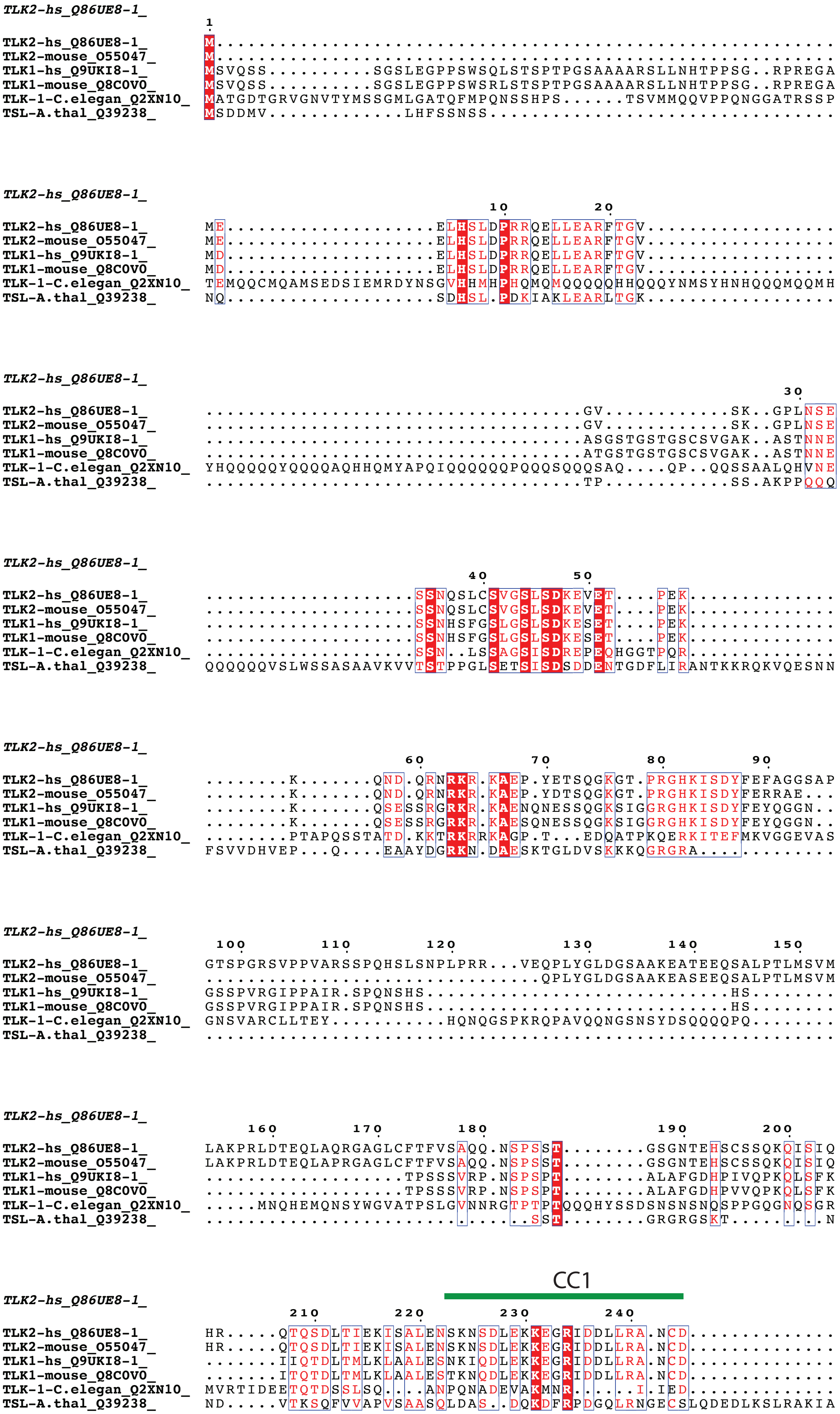

Supplementary Figure 1. TLKs sequence alignment. Sequence alignment of TLK2 from plants to human showing a highly-conserved protein sequence around the predicted coiled-coil regions and the kinase domain. Red box and white residue symbolise strict identity conservation. Red residue symbolises similar physicochemical properties. Blue frame symbolises similarity across the group.

(Section B)

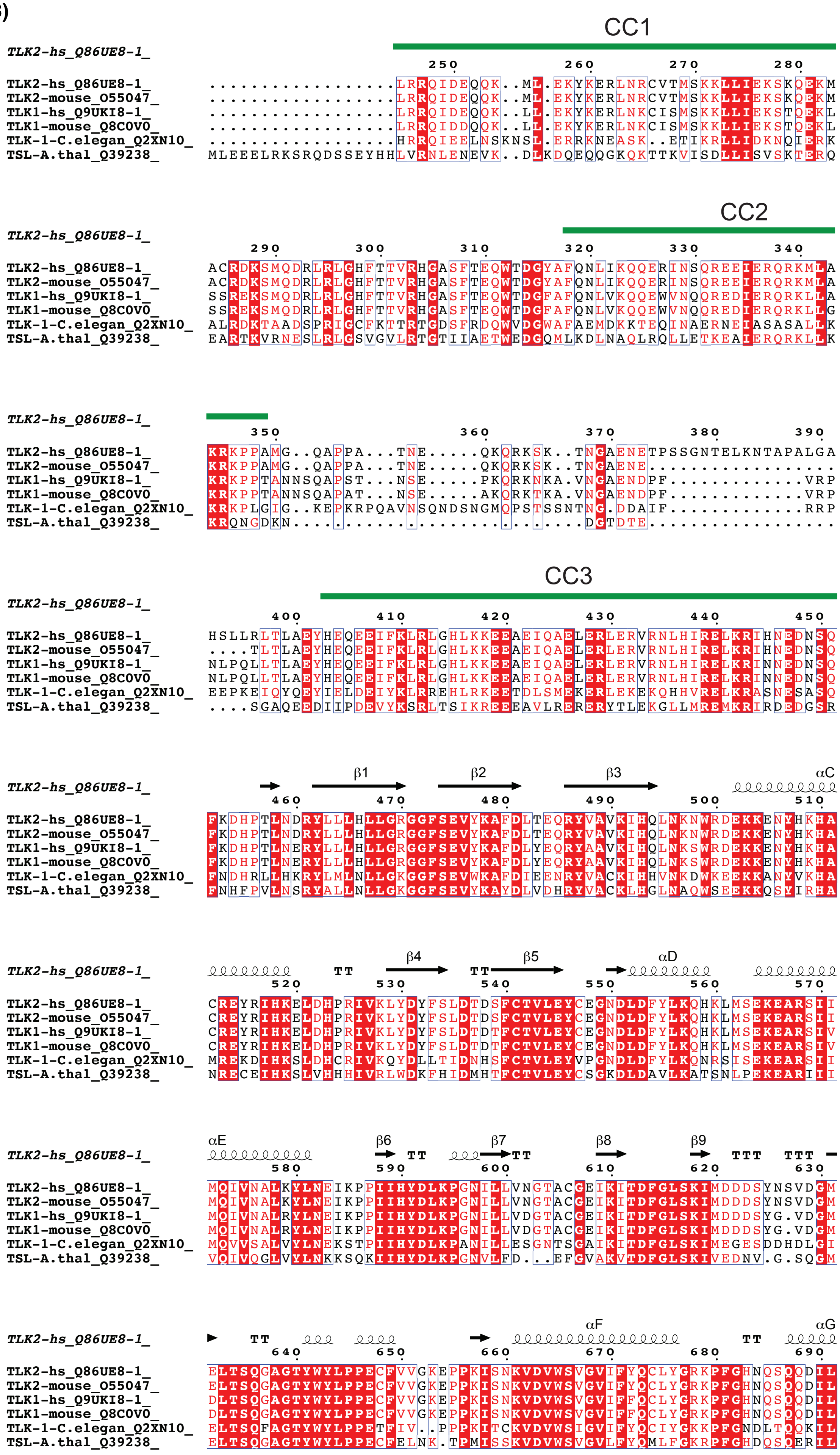

Supplementary Figure 1. TLKs sequence alignment. Sequence alignment of TLK2 from plants to human showing a highly-conserved protein sequence around the predicted coiled-coil regions and the kinase domain. Red box and white residue symbolise strict identity conservation. Red residue symbolises similar physicochemical properties. Blue frame symbolises similarity across the group.

(Section C)

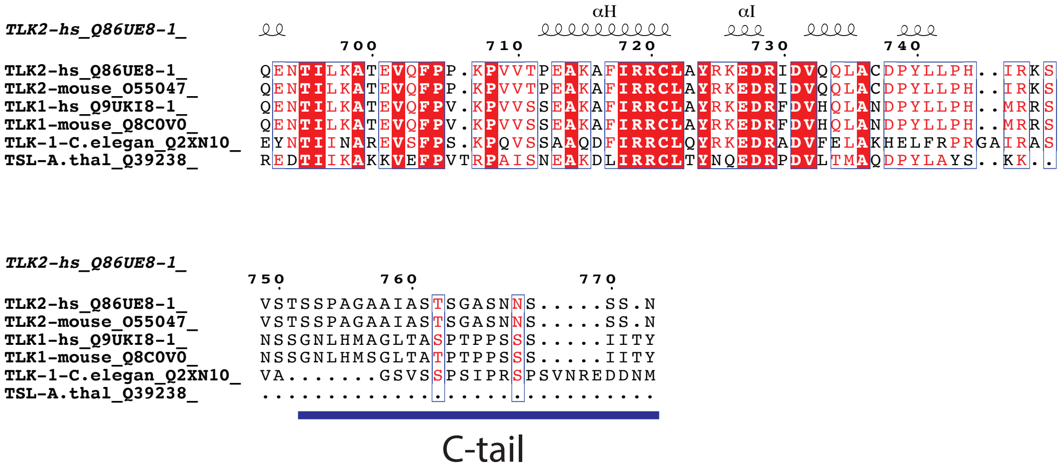

Supplementary Figure 1. TLKs sequence alignment. Sequence alignment of TLK2 from plants to human showing a highly-conserved protein sequence around the predicted coiled-coil regions and the kinase domain. Red box and white residue symbolise strict identity conservation. Red residue symbolises similar physicochemical properties. Blue frame symbolises similarity across the group.

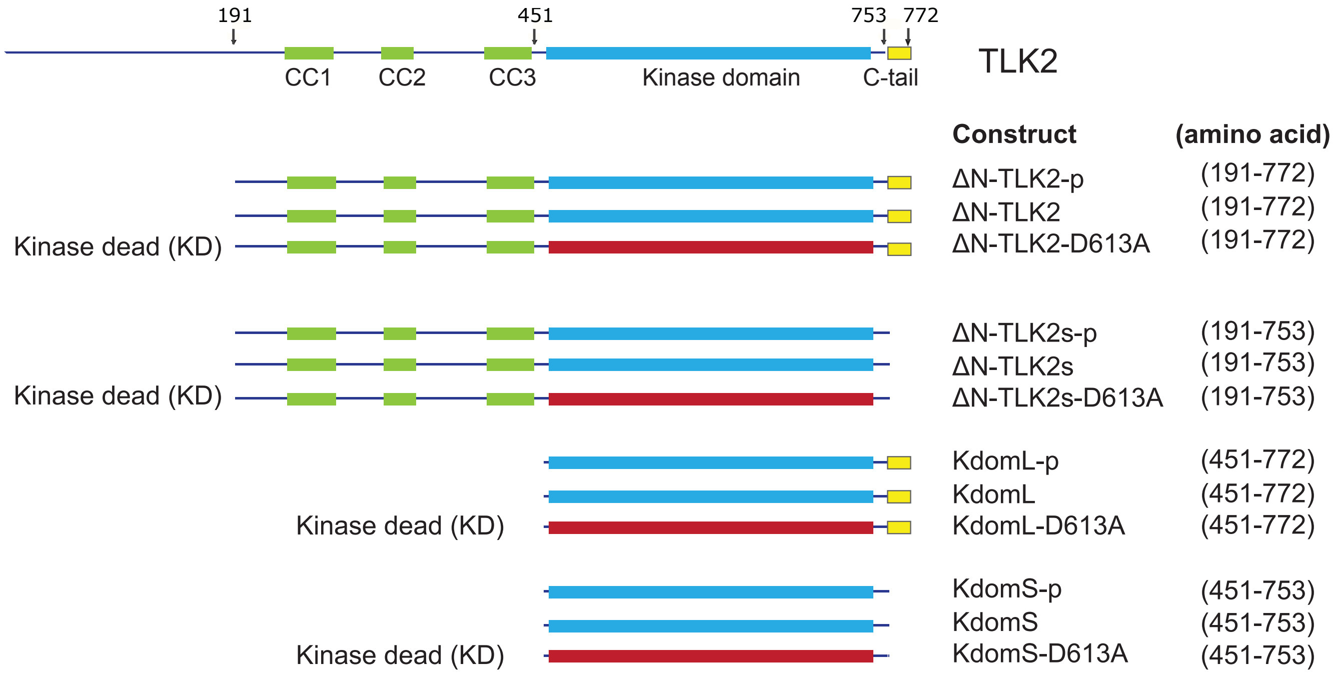

Supplementary Figure 2. Detailed scheme of the constructs used in this study. a) TLK2 Domain architecture and constructs. All the constructs were overexpressed, purified and validated by mass spectrometry. Coiled-coil domains (Green), wild-type kinase domain (Blue), kinase-dead domain D613A (Red), and C-tail (Yellow).

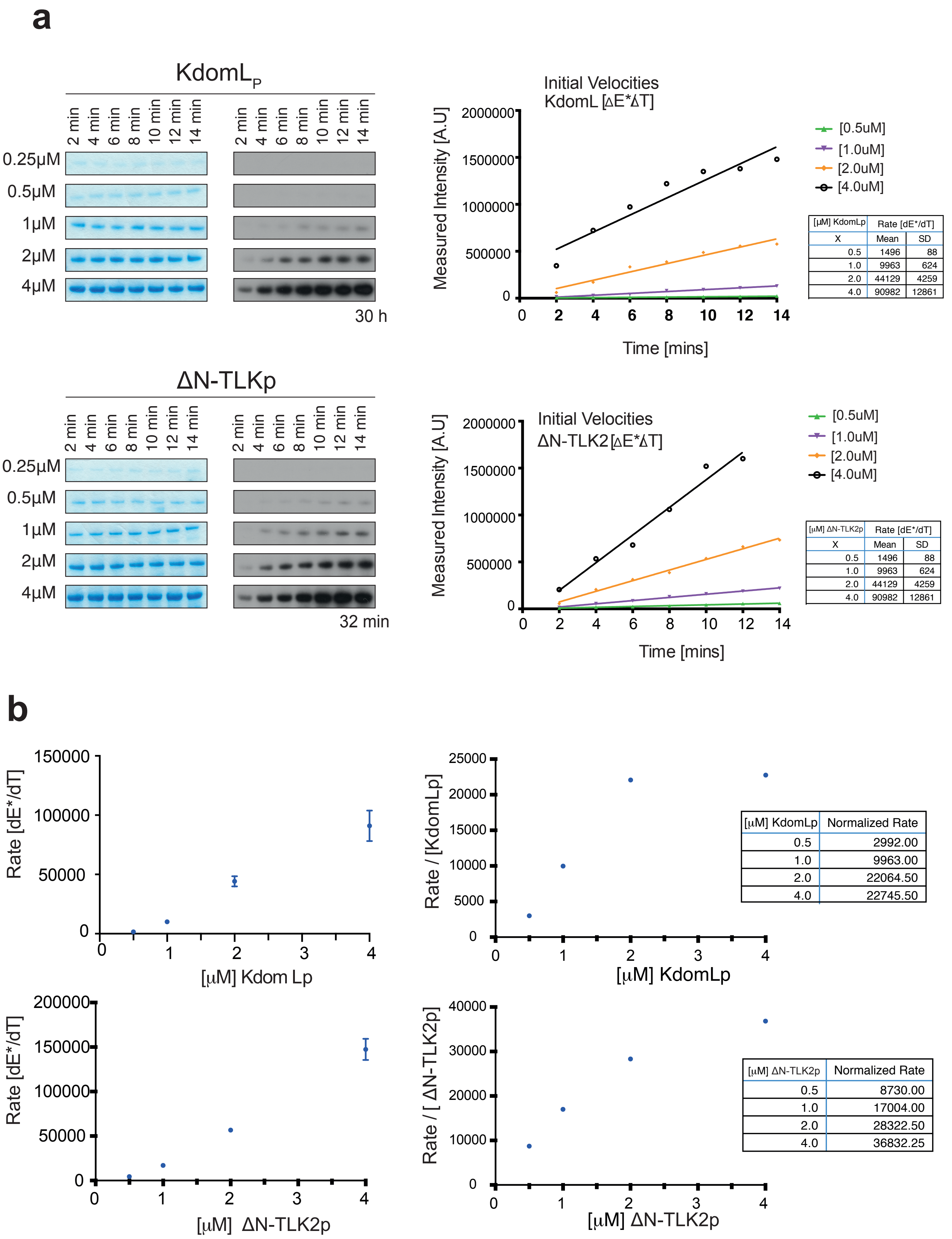

Supplementary Figure 3. ΔN-TLK2 and Kinase domain kinetics. a) SDS-PAGE and autoradiograms (left panels) together with the initial velocity for ΔN-TLK2-p and its respective kinase domain (KdomLp) calculated via densitometric measurements (right panels). b) Plots showing autophosphorylation rates. The tables show the autophosphorylation rates normalized with the protein concentration present in each measurement (mean ± s.d., $n = 3$ biological replicates).

a

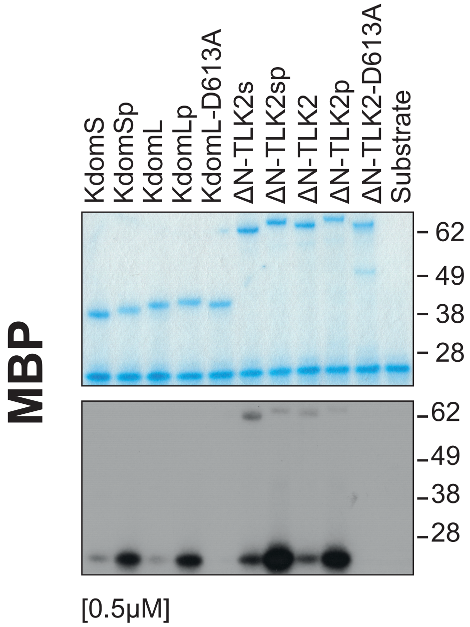

b

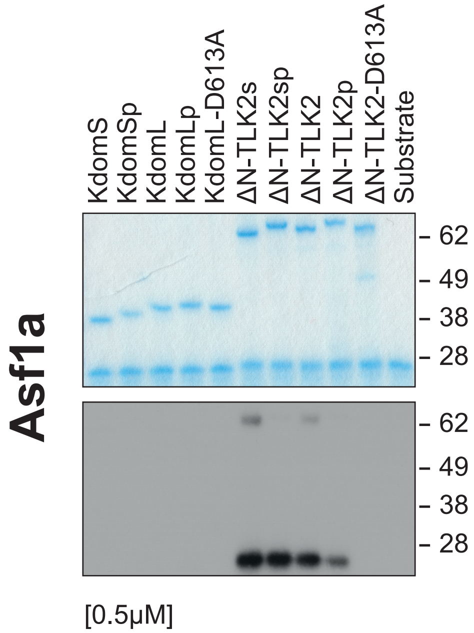

Supplementary Figure 4. Phosphorylation of TLK2 substrates. SDS-PAGE and autoradiograms of substrate phosphorylation by various TLK2 constructs showing a) MBP and b) ASF1a phosphorylation.

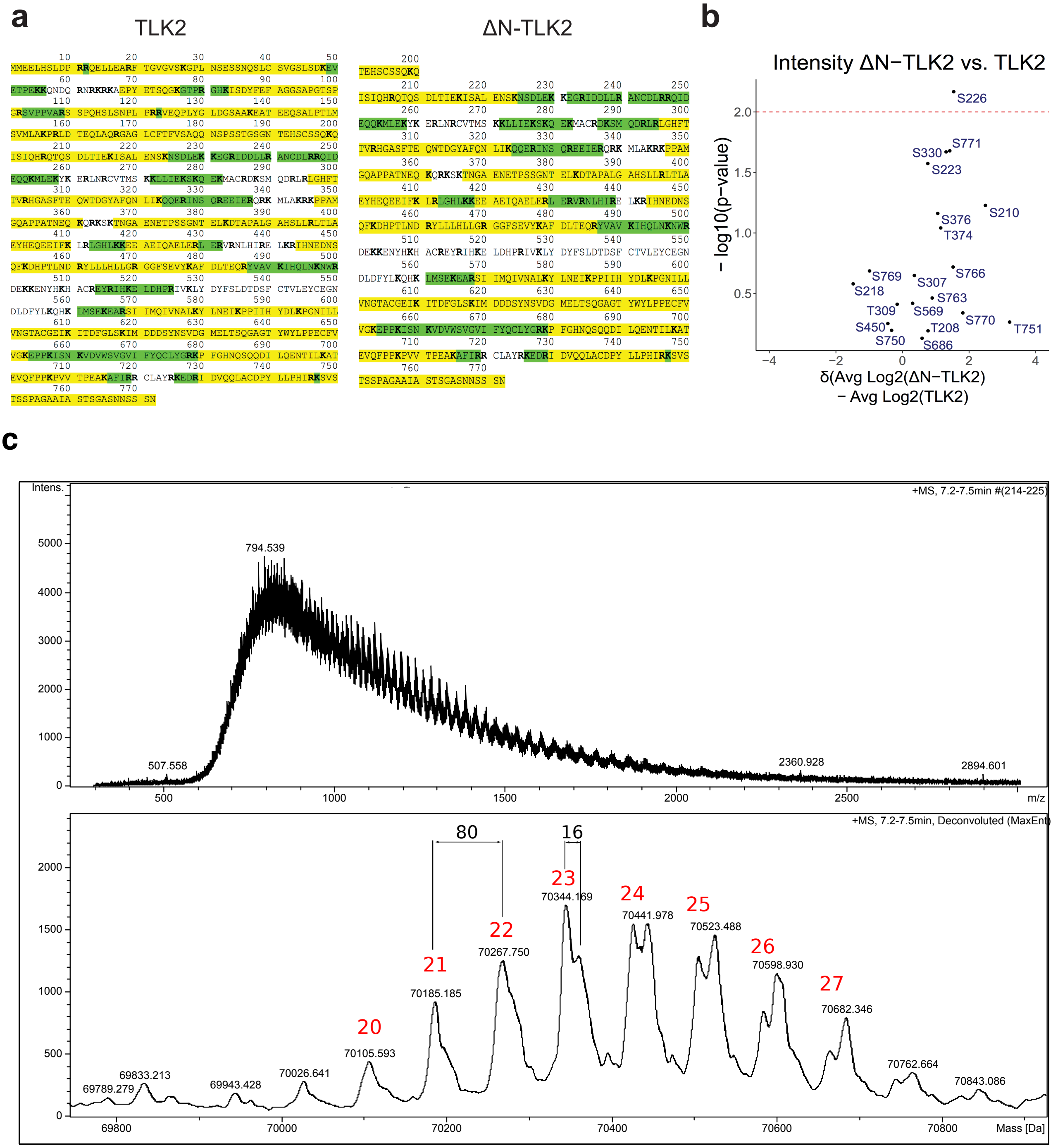

Supplementary Figure 5. Mass spectrometry analysis of TLK2 and ΔN-TLK2. a) Overview of the TLK2 mass spectrometry peptide coverage after expression in HEK293 cells. The tryptic coverage is marked in yellow, while miss-cleaved peptides are shown in green These are a by-product of the tryptic protein digest and usually not as abundant as the tryptic peptides. b) Differences in phosphorylation between TLK2 and ΔN-TLK2 constructs expressed in HEK293 cells. The heatmap contains normalized log-transformed intensities for all 3 replicates of the two conditions. The analysis shows a t-test with this dataset and attached the resulting Volcano plot. According to this, sites S226, S771, S330, and S223 are significantly (p = 0.05) higher in phosphorylation in the truncated construct. c) Positive ion ESI-TOF mass spectrum (top) and the deconvoluted spectrum (bottom) of intact ΔN-TLK2 sample used in Fig 3. The number of phosphorylations are labelled in red. The shoulder peaks, which become the dominant species at higher phosphorylation states have an added mass of 16 Da, which is likely due to oxidation of a methionine residue.

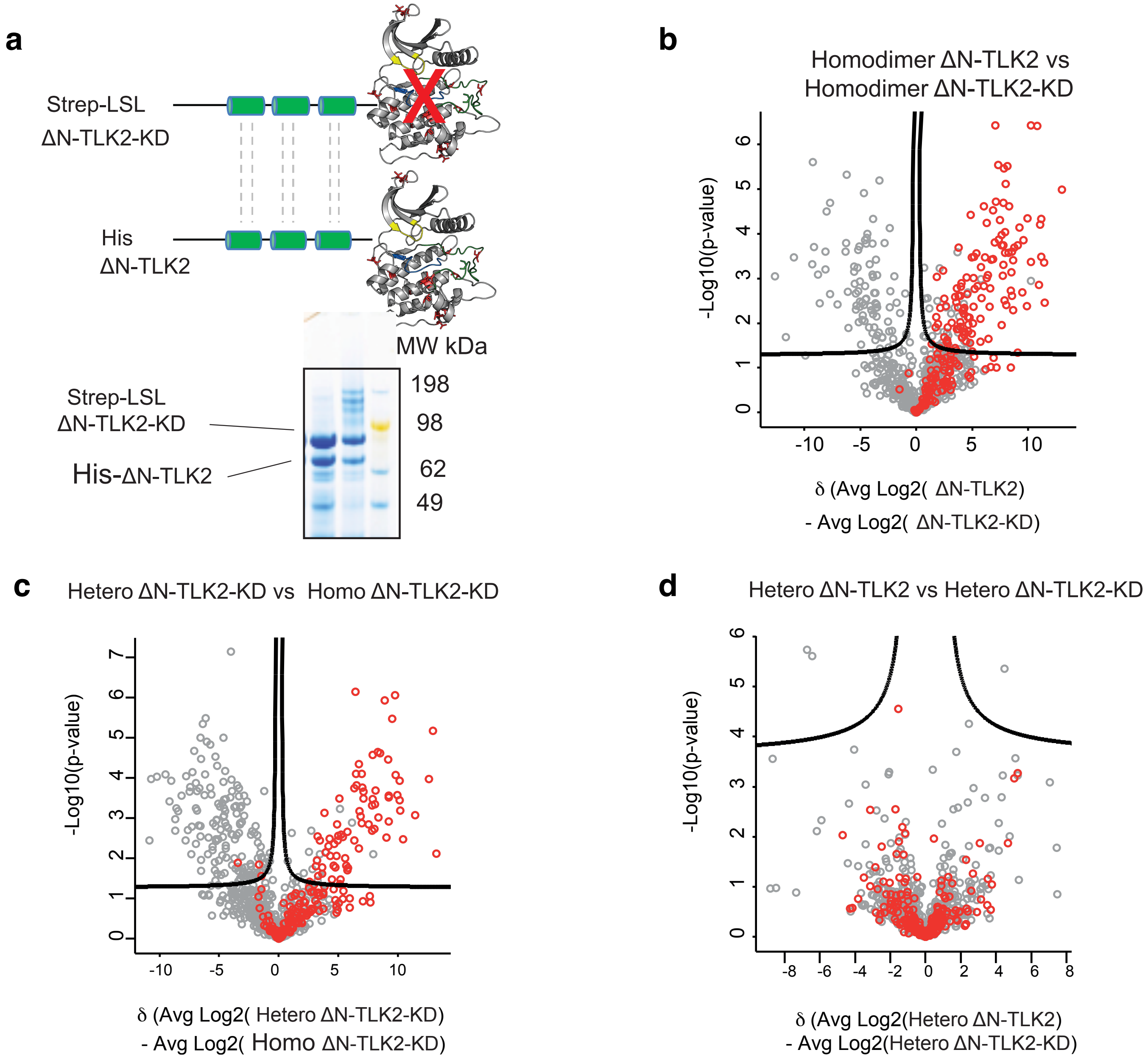

Supplementary Figure 6. Mass spectrometry analysis of TLK2, ΔN-TLK2 and ΔN-TLK2 heterodimer. a) Schematic representation of a fusion TLK2 construct with two different tags, Twin-Strep-LSL tag on the kinase-dead and a penta-His tag on a kinase active protein. SDS-PAGE showing the two proteins, Twin-Strep-LSL-ΔN-TLK2-KD dead and penta-His-ΔN-TLK2 that were separated and extracted for trypsin digest and MS analysis. b) Volcano plot based on t-test analysis showing a clear difference in phosphopeptides (marked in red) detected in the ΔN-TLK2 and ΔN-TLK2-KD homodimers. c) Volcano plot based on t-test analysis comparing the ΔN-TLK2-KD phosphopeptides in the context of the heterodimer and the homodimer. d) Volcano plot based on t-test analysis comparing the phosphopeptides of ΔN-TLK2 and ΔN-TLK2-KD in the context of the heterodimer.

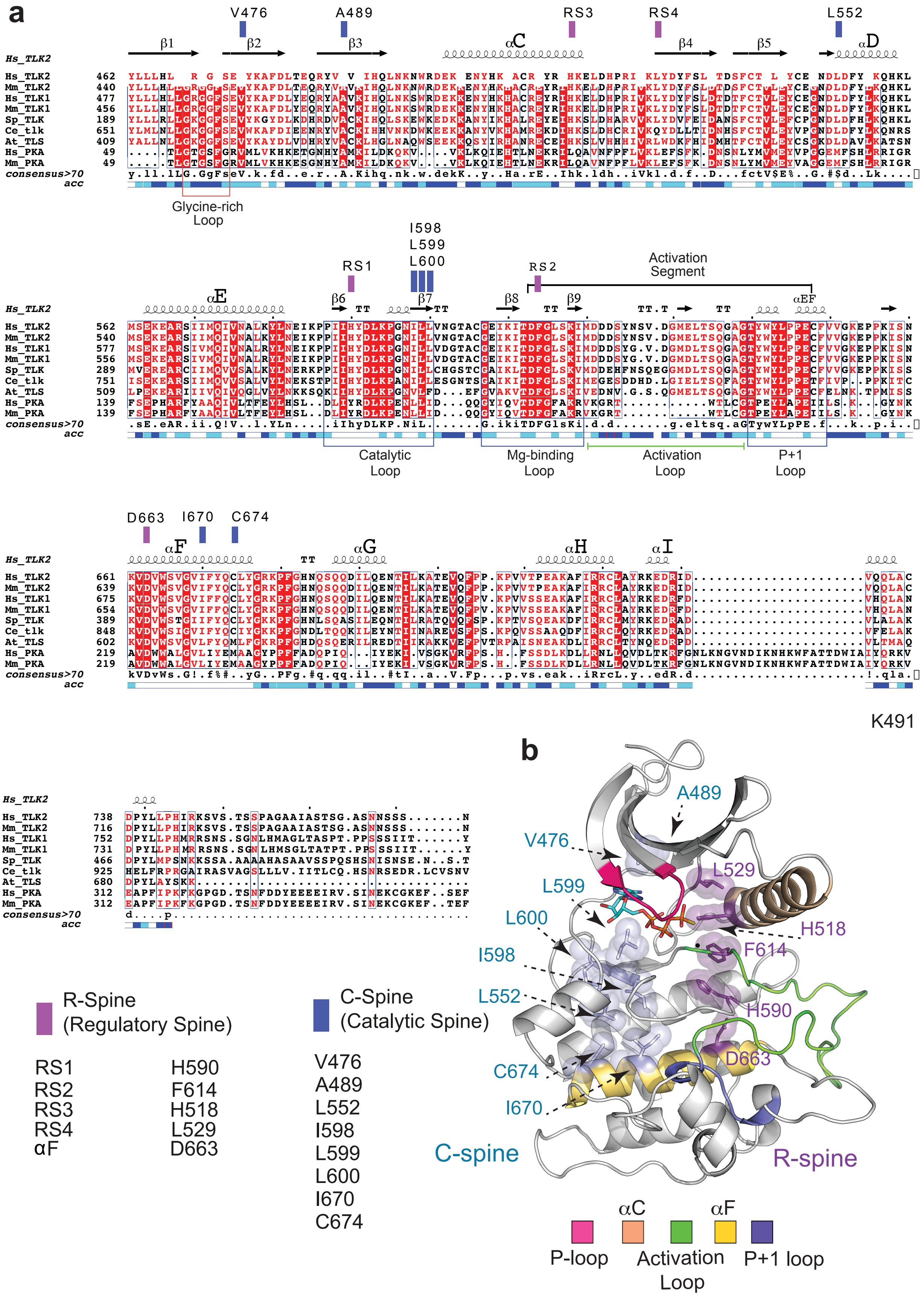

Supplementary Figure 7. TLK2 kinase domain regulatory segments. a) Sequence alignment of TLK kinase domains from various species and the human PKA protein kinase. All the important elements in the kinase domain such as activation loop, activation segment, catalytic loop, glycine-rich region, Magnesium-binding loop, R-spine and C-spine are highlighted. Localization of the TLK2 residues presumably involved in the formation of the R-spine, and the C-spine was based on the localization of those residues in PKA. Localization of the important elements of the kinase domain according to Ref [48] . Red box and white residue symbolise strict identity conservation. Red residue symbolises similar physicochemical properties. Blue frame symbolises similarity across the group. b) Mapping of the R- and C- spines in the TLK2 kinase domain structure.

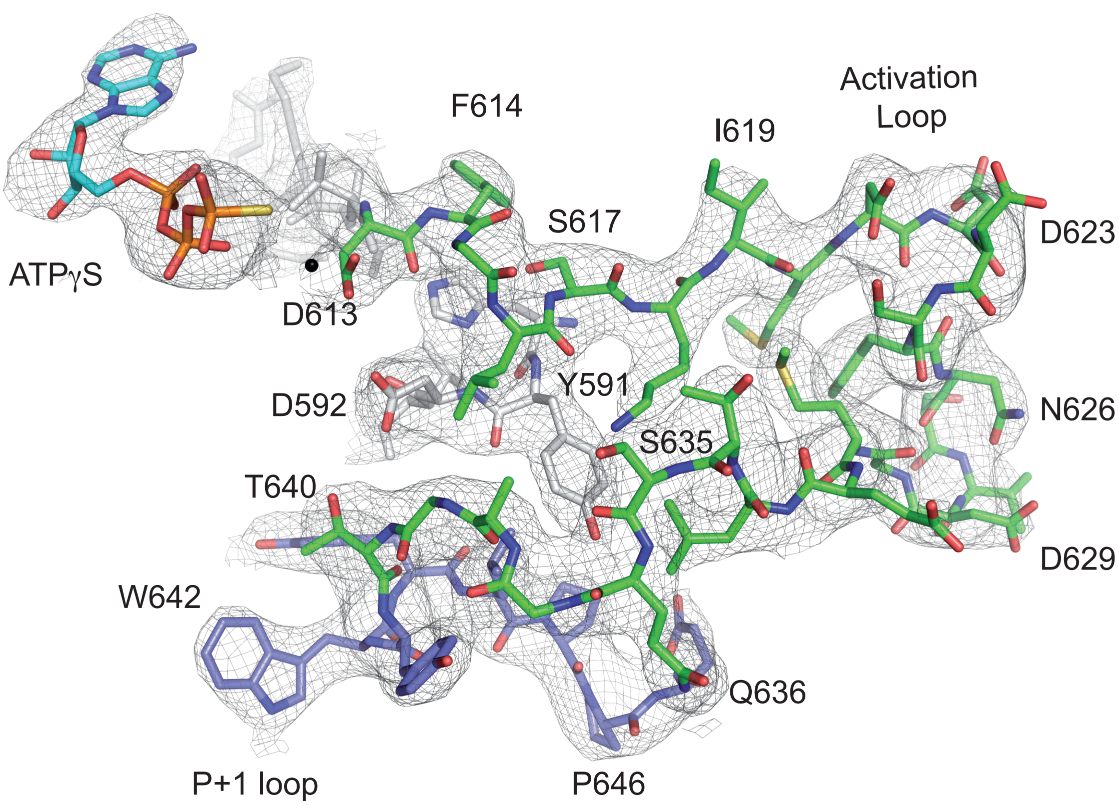

Supplementary Figure 8. Detailed view of the electron density map of the TLK2 kinase structure. The characteristic region of the TLK family containing the activation loop and a section of the P+1 loop is shown. The figure displays the 2mFo-DFc refined electron density map contoured at 1.2 σ.

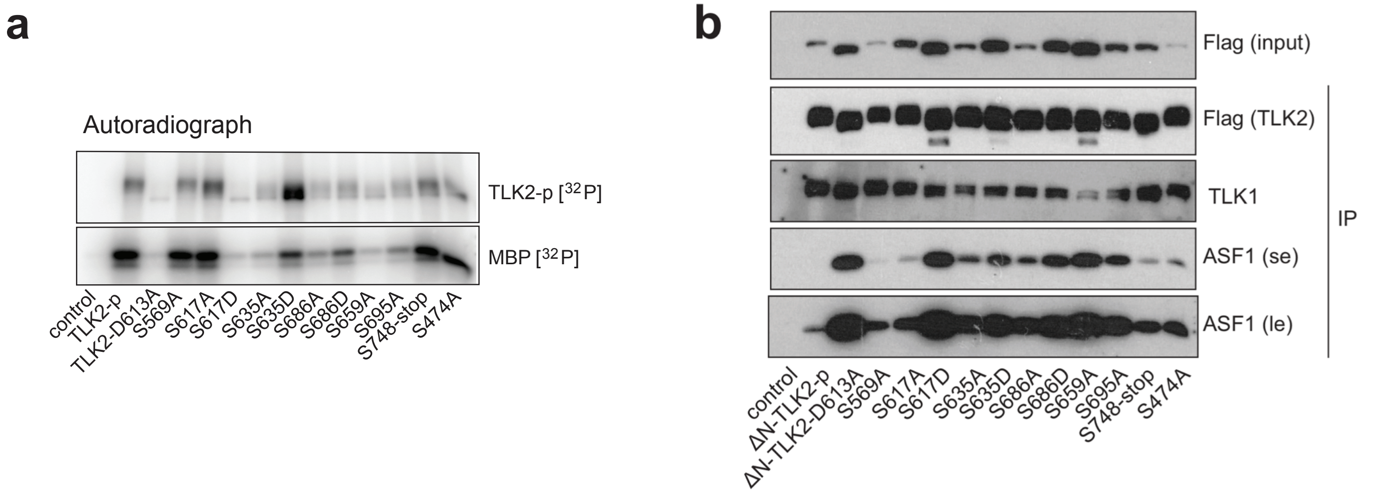

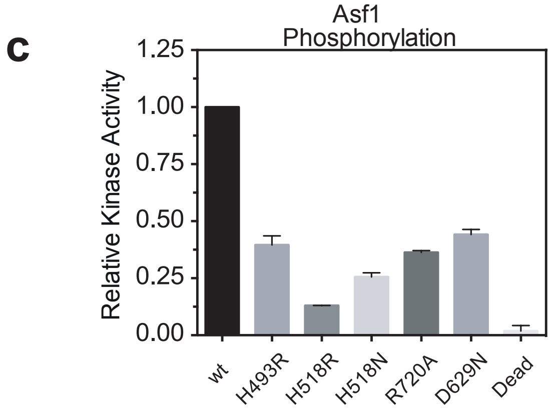

ΔN-TLK2-p

	wt	H493R	H518R	H518N	R720A	D629N	Dead
Mean	1.000	0.3955	0.1299	0.2557	0.3633	0.4413	0.01834
Std. Deviation	0.0	0.04001	0.0003931	0.01783	0.008127	0.02256	0.02390

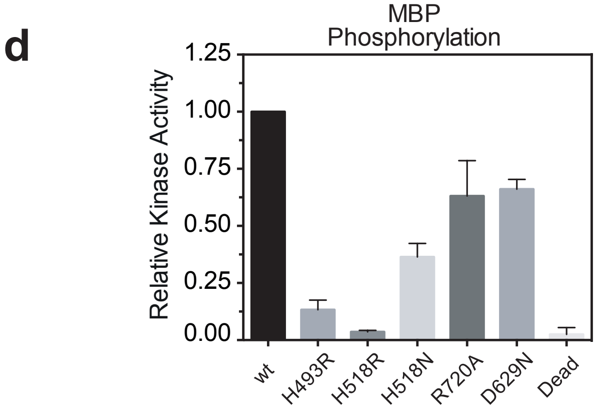

ΔN-TLK2-p

	wt	H493R	H518R	H518N	R720A	D629N	Dead
Mean	1.000	0.1331	0.03623	0.3638	0.6307	0.6606	0.02489
Std. Deviation	0.0	0.04188	0.006270	0.05919	0.1551	0.04327	0.03006

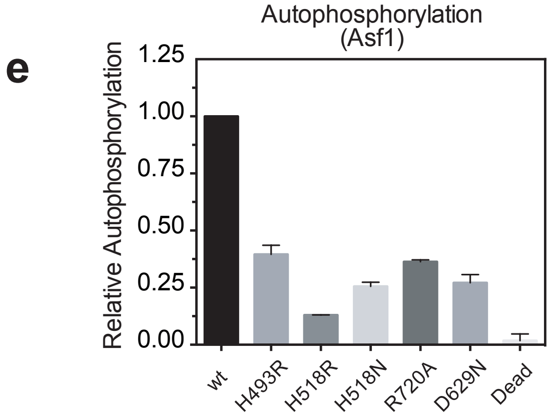

ΔN-TLK2-p

	wt	H493R	H518R	H518N	R720A	D629N	Dead
Mean	1.000	0.3955	0.1299	0.2557	0.3633	0.2710	0.01771
Std. Deviation	0.0	0.04001	0.0003931	0.01783	0.008127	0.03596	0.02944

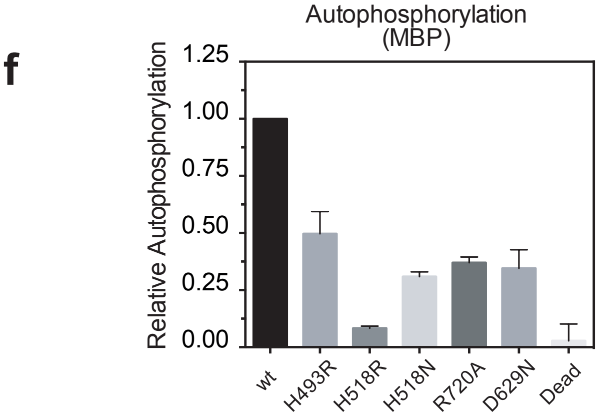

ΔN-TLK2-p

	wt	H493R	H518R	H518N	R720A	D629N	Dead
Mean	1.000	0.4963	0.08276	0.3088	0.3691	0.3455	0.02772
Std. Deviation	0.0	0.09780	0.009417	0.02095	0.02565	0.08103	0.07421

Supplementary Figure 9. Activity assays for different TLK2 mutants. a) Representative autoradiograms of *in vitro* kinase assays of Strep-pulldowns from cells expressing Strep-FLAG tagged TLK2 phosphorylation site mutants (S>A) or mimics (S>D). Results are quantified in Fig. 5b. b) Western blotting of input or Strep-pulldowns from AD293 cells transiently expressed with Strep-FLAG tagged TLK2 mutants. Levels of co-purified ASF1 are shown. Short or long exposure of the same film is indicated as (se) or (le), respectively. Histograms showing ID mutants phosphorylation of c) ASF1a and d) MBP. Kinase autophosphorylation profiles of the ID mutants when using e) ASF1a and f) MBP as substrates. The data points indicate the relative kinase activity and the autophosphorylation activity of the TLK2-mutants normalized to the activity of the wild-type protein (mean ± s.d., $n = 3$ biological replicates).

Figure 1D-E: Western (D) and autoradiographs (E)

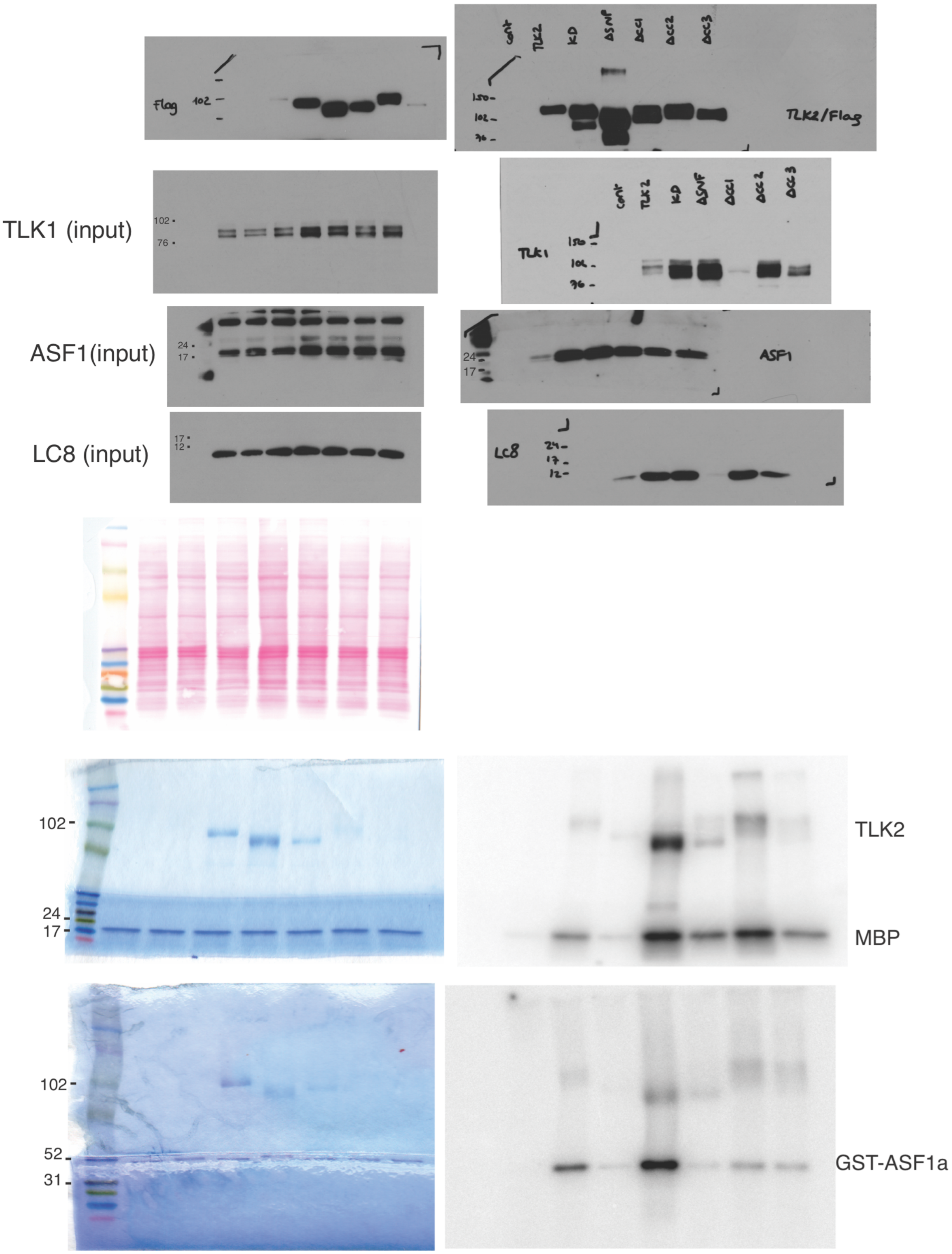

Supplementary Figure 10. Uncropped gels for figure 1D and 1E

Figure 2a

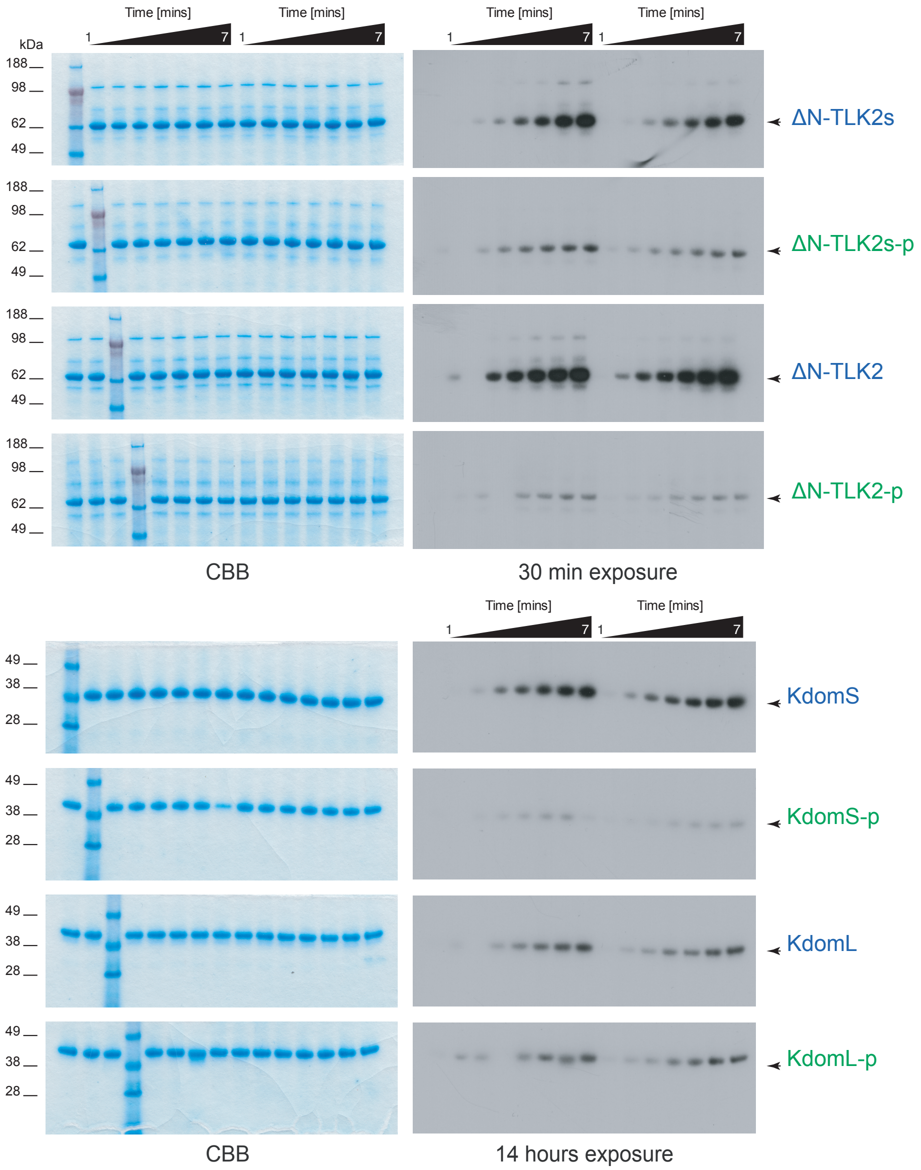

Figure 2c

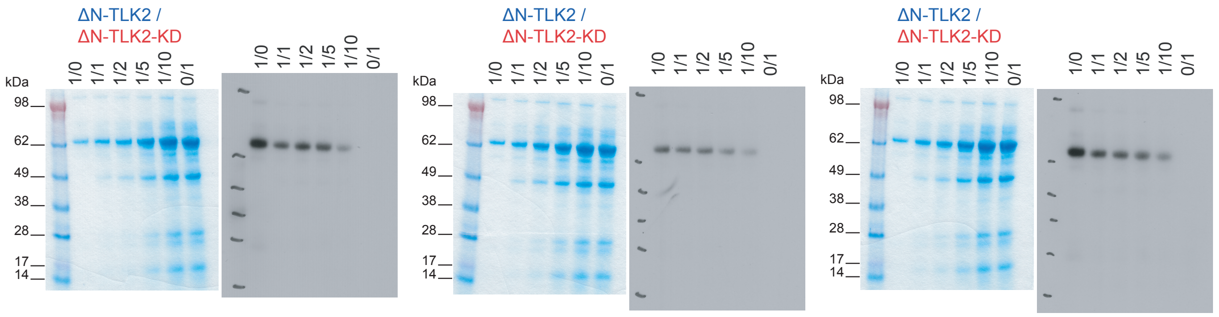

Supplementary Figure 11. Uncropped gels for figure 2a and 2c

Figure 5d

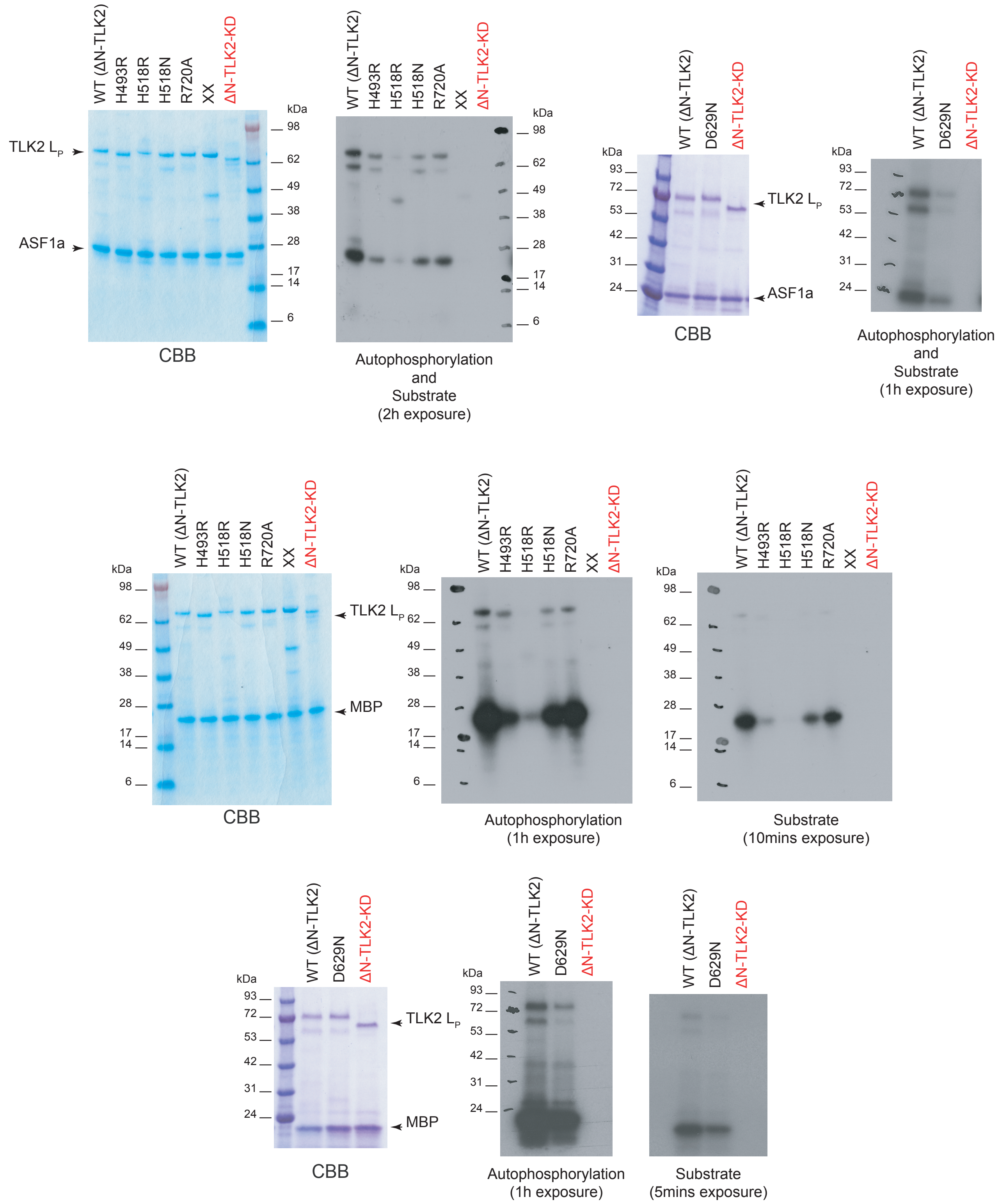

Supplementary Figure 12. Uncropped gels for figure 5d

Figure 6A: autoradiographs and Coomassie

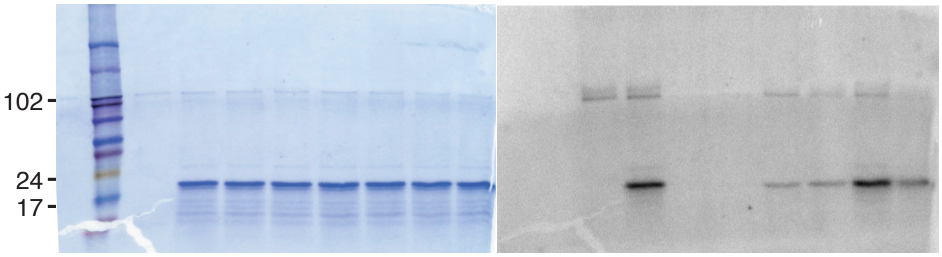

Supplementary Figure 13. Uncropped gels for Figure 6A autoradiograph and coomassie

Supplementary Figure 3a

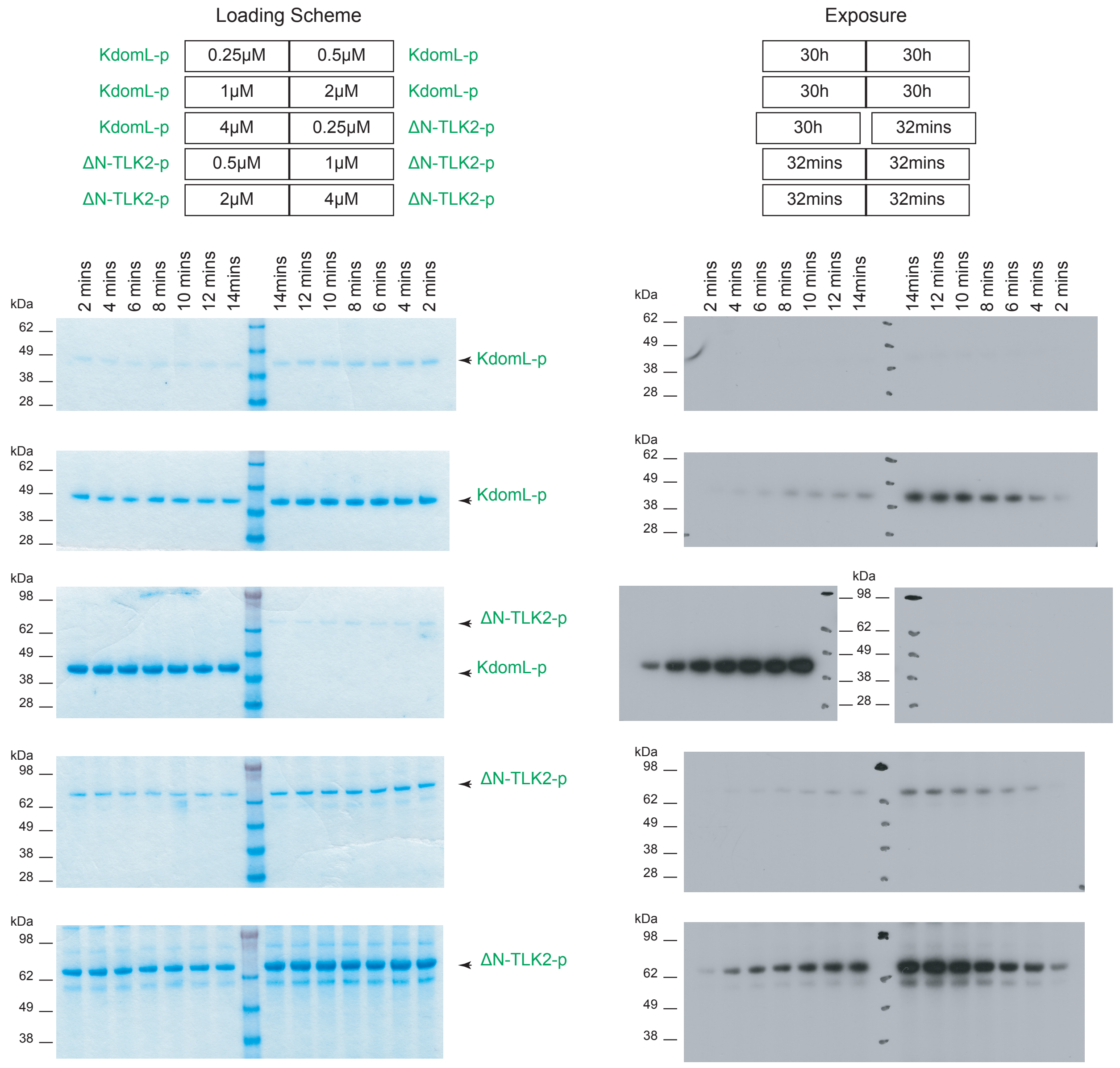

Supplementary Figure 14. Uncropped gels for Supplementary figure 3a

Supplementary Figure S8A-B: autoradiographs (A) and Western (B)

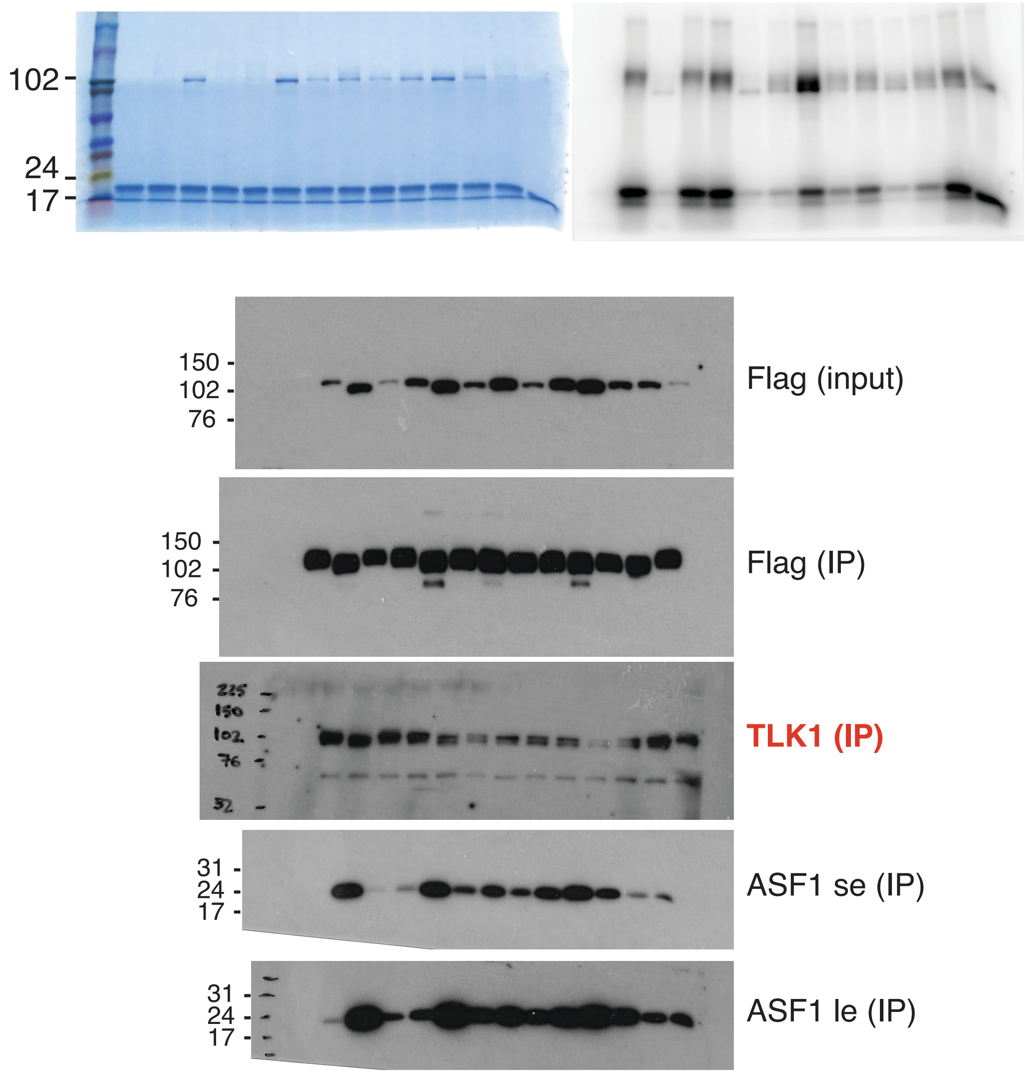

Supplementary Figure 15. Uncropped gels for Supplementary figure 8a-b

Construct	TLK2 primer name	DNA sequence
	InFusion Cloning	
	Vector fwd	gcttgcggccgcataatgcttaag
	Vector rev	ctggctgtggtgatgatggtgat
KdomS (451-753)	KdomS fwd	catcaccacagccagGATCCACAATTT
	KdomS rev	tatgcggccgcaagctTTTAGCTACTT
KdomL (451-772)	KdomL fwd	catcaccacagccagCAATTTAAAGATCATCCAACGCTAAATGACAGA
	KdomL rev	tatgcggccgcaagcTCAATTAGAAGAACTGTTATTGGACGCCC
ΔN_TLK2 (191-772)	ΔN_TLK2 fwd	catcaccacagccagACAGAGCATTCCTGCAGCTCCC
	ΔN_TLK2 rev	tatgcggccgcaagcTCAATTAGAAGAACTGTTATTGGACGCCC
ΔN_TLK2s (191-753)	ΔN_TLK2s fwd	catcaccacagccagACAGAGCATTCCTGCAGCTCCC
	ΔN_TLK2s rev	tatgcggccgcaagctTTTAGCTACTT
	SDM primer name	DNA sequence
D613A	D613A_Fwd	GTGGAGAGATAAAAATTACAGcaTTTGGTCTTTCGAAGATCATGGATGATG
	D613A_Rev	CATCATCCATGATCTTCGAAAGACCAAAtgCTGTAATTTTTATCTCTCCAC
H493R	H493R_Fwd	TGTGAAAATTcgcCAGTTAAATAAAAAC
	H493R_Rev	GCTACGTATCTTTGCTCTG
H518R	H518R_Fwd	ATACCGGATTcgcAAAGAGCTGGATC
	H518R_Rev	TCCCTACATGCATGCTTG
H518N	H518N_Fwd	ATACCGGATTaacAAAGAGCTGGATC
	H518N_Rev	TCCCTACATGCATGCTTG
R720A	R720A_Fwd	GTTTATTCGAgccTGCTTGGCCTACCGAAAG
	R720A_Rev	GCCTTTGCTTCAGGTGTT
D629N	D629N_Fwd	CAATTCAGTGaacGGCATGGAGC
	D629N_Rev	TAGCTATCATCATCCATG

Supplementary Table 1. TLK2 Primer sequences. All SDM were carried out using Q5 SDM Kit (NEB) except for D613A that was performed using QuickChange II Kit (Agilent). The lambda phosphatase was cloned into MCS2 of a pET_Duet vector using the traditional restriction enzymes cloning (BamHI and HindIII).

1st G-Block Codes for Twin-Strep_LSL and a part of TLK2
CATATGatggatagcgcttggagccacccgcagttcgagaaaggtggaggttccggaggtggatcgggaggtggatcg<br>tggagccacccgcagttcgaaaaaggcgccagcggtgtagatctgggtaccATGACCGACATCTACATCCCGCCGGAG<br>GGTCTCTACTTCCGCCTCCTTGGCTTTGCCAGTCGGCAGGTGATCTTCGCGCGCAACTCTCCCTCTCCCGATGTTGGT<br>CTGTCTCCGGTCAACGACCAGGCTACCGACCAGTACTTCTCGCTCATCTACGGCACTGGAGAACACGCCGGTCTCTAC<br>GCGATAAAGAGCAAAGCGACGGGCAAGGTGCTCTTCTCGCGTAGGCCTGCGGAACCGTATGTGGGCCAAATCGATGGC<br>GACGGGCGTTATCCCGACAACTGGTTCAAGATTGAGCCAGGAAAGACCTATCTCTCCAAATATTTCCGGCTCGTTCAG<br>CCGTCGACTGGCACCGCGCTTGTCTCGCGCACGCATTTGCAGCCATACTTCTGGAATCACCCTCAGACTGAAGTCTTC<br>GACGACCAATACTTCACCTTCCTCTTCGAGGATgagaacctgtacttccaatccACAGAGCATTCCTGCAGCTCCCAA<br>AAACAGATCTCCATCCAGCACAGACAGACCCAGTCCGACCTCACAATAGAAAAAATATCTGCACTAGAAAACAGTAAG<br>AATTCTGACTTAGAGAAGAAGGAGGGAAGAATAGATGATTTATTAAGAGCCAACTGTGATTTGAGACGGCAGATTGAT<br>GAACAGCAAAAGATGCTAGAGAAATACAAGGAACGATTAAATAGATGTGTGACAATGAGCAAGAAACTCCTTATAGAA<br>AAGTCAAAACAAGAGAAGATGGCGTGTAGAGATAAGAGCATGCAAGACCGCTTGAGACTGGGCCACTTTACTACTGTC<br>CGACACGGAGCCTCATTTACTGAACAGTGGACAGATGGTTATGCTTTTCAGAATCTTATCAAGCAACAGGAAAGGATA<br>AATTCACAGAGGGAAGAGATAGAAAGACAACGGAAAATGTTAGCAAAGCGGAAACCTCCTGCCATGG
2nd G-Block code for the rest of the TLK2 protein (with the D613A mutation)
CCATGGGTCAGGCCCCTCCTGCAACCAATGAGCAGAAACAGCGGAAAAGCAAGACCAATGGAGCTGAAAATGAAACGC<br>CCTCTTCTGGGAATACAGAGCTAAAGGATACAGCCCCAGCCTTAGGAGCCCACAGTTTACTTAGGTTAACGTTAGCAG<br>AATACCATGAACAAGAAGAAATCTTCAAACTCAGATTAGGTCATCTTAAAAAGGAGGAAGCAGAGATCCAGGCAGAGC<br>TGGAGAGACTAGAAAGGGTTAGAAATCTACATATCAGGGAACTAAAAAGGATACATAATGAAGATAATTCACAATTTA<br>AAGATCATCCAACGCTAAATGACAGATATTTGTTGTTACATCTTTTGGGTAGAGGAGGTTTCAGTGAAGTTTACAAGG<br>CATTTGATCTAACAGAGCAAAGATACGTAGCTGTGAAAATTCACCAGTTAAATAAAAACTGGAGAGATGAGAAAAAGG<br>AGAATTACCACAAGCATGCATGTAGGGAATACCGGATTCATAAAGAGCTGGATCATCCCAGAATAGTTAAGCTGTATG<br>ATTACTTTTCACTGGATACTGACTCGTTTTGTACAGTATTAGAATACTGTGAGGGAAATGATCTGGACTTCTACCTGA<br>AACAGCACAAATTAATGTCGGAGAAAGAGGCCCGGTCCATTATCATGCAGATTGTGAATGCTTTAAAGTACTTAAATG<br>AAATAAAACCTCCCATCATACACTATGACCTCAAACCAGGTAATATTCTTTTAGTAAATGGTACAGCGTGTGGAGAGA<br>TAAAAATTACAGCGTTTGGTCTTTCGAAGATCATGGATGATGATAGCTACAATTCAGTGGATGGCATGGAGCTAACAT<br>CACAAGGTGCTGGTACTTATTGGTATTTACCACCAGAGTGTTTTGTGGTTGGGAAAGAACCACCAAAGATCTCAAATA<br>AAGTTGATGTGTGGTCGGTGGGTGTGATCTTCTATCAGTGTCTTTATGGAAGGAAGCCTTTTGGCCATAACCAGTCTC<br>AGCAAGACATCCTACAAGAGAATACGATTCTTAAAGCTACTGAAGTGCAGTTCCCGCCAAAGCCAGTAGTAACACCTG<br>AAGCAAAGGCGTTTATTCGACGATGCTTGGCCTACCGAAAGGAGGACCGCATTGATGTCCAGCAGCTGGCCTGTGATC<br>CCTACTTGTTGCCTCACATCCGAAAGTCAGTCTCTACAAGTAGCCCTGCTGGAGCTGCTATTGCATCAACCTCTGGGG<br>CGTCCAATAACAGTTCTTCTAATTGACTCGAG

Supplementary Table 2. Cloning strategy for heterodimer ΔTLK2- ΔTLK2 KD

The plasmid containing 6xHis_ΔN_TLK2 was digested with NdeI and XhoI (MCS2 of pET_Duet)
Two inserts obtained from G-Blocks were then ligated after restriction enzyme digests:
1st G-Block was digested with NdeI / NcoI and the 2nd G-Block was digested with NcoI /XhoI to produce a heterodimer (6xHis_ΔN_TLK2 and TwinStrepTAG_LSL_ΔN_TLK2_KD).

Lysis Buffer	20mM Tris, 500mM NaCl, 20mM Imidazole, 0.2 mM TCEP, pH8.8, protease inhibitor (1/50ml)
HisTrap Chealting HP (5ml) Buffer A (Binding buffer)	20mM Tris, 500mM NaCl, 20mM Imidazole, 0.2 mM TCEP,
HisTrap Chealting HP (5ml) Buffer B (Elution buffer)	20mM Tris, 25mM NaCl, 500mM Imidazole, 0.2 mM TCEP, pH8.8
HiTrap Q HP (1ml) Buffer A (Binding buffer)	20mM Tris, 25mM NaCl, 0.2 mM TCEP, pH8.8
HiTrap Q HP (1ml) Buffer A (Elution buffer)	20mM Tris, 1.0 M NaCl, 0.2 mM TCEP, pH8.8
Superdex 75 16/60 SEC Buffer	20mM HEPES, 150 mM NaCl, 0.2 mM TCEP, pH7.5

Supplementary Table 3. TLK Purification buffers.
