# Supplementary material for: Molecular basis of Tousled-Like Kinase 2 activation
Source: Nat Commun. 2018 Jun 28;9:2535. doi: 10.1038/s41467-018-04941-y (PMC6023931; doi:10.1038/s41467-018-04941-y)
Supplement: Supplementary file 3 — Description of Additional Supplementary Files [file 41467_2018_4941_MOESM3_ESM.pdf]

Description of Additional Supplementary Files

File Name: Supplementary Data 1
Description:
1.- for the HEK293 expressed TLK2 constructs phosphosite table: Phosphorylation sites identified by LC-MS/MS in TLK2 expressed in HEK293 cells.
Sheet 1 “Phospho(STY)Sites” contains the raw-output of the MaxQuant search output.
Sheet 2 “P(STY) Sites TLK2” is a subset of sheet 1 with only sites in TLK2.
Sheet 3 “TLK2 Norm P-Int” shows the log-transformed and normalized (row-wise median subtraction) intensities of all phosphorylation sites.
Sheet 4 “TLK2 Median Occupancy” contains occupancy values for all phosphorylation sites represented with median and standard deviation per construct.
2.- for the E.coli expressed ΔN-TLK2 phosphosite table: Phosphorylation sites identified by LC-MS/MS in TLK2 expressed in E. coli.
Sheet 1 “Phospho(STY)Sites” contains the raw-output of the MaxQuant search output.
Sheet 2 “P(STY) Sites TLK2” is a subset of sheet 1 with only sites in TLK2.
Sheet 3 “TLK2 Filtered-41Sites NormInt” contains the log-transformed and normalized (row-wise median subtraction) intensities of all phosphorylation sites with missing values imputed as specified in the supplementary methods.
Sheet 4 “NormInt Distribution” confirms the normal distribution of the intensity values.
Sheet 5 “KDom Filter-11Sites NormInt M41” shows only phosphorylation sites in the kinase-domain.
